# Oxidative Stress Induced Damage and Early Senescence in Preterm Placenta

**DOI:** 10.1155/2021/9923761

**Published:** 2021-06-24

**Authors:** Yudianto Budi Saroyo, Noroyono Wibowo, Rima Irwinda, Ani Retno Prijanti, Evy Yunihastuti, Saptawati Bardosono, Sofie Rifayani Krisnadi, Putri Indah Permata, Stephanie Wijaya, Victor Prana Andika Santawi

**Affiliations:** ^1^Maternal Fetal Division, Department of Obstetrics and Gynecology, Faculty of Medicine Universitas Indonesia/Cipto-Mangunkusumo Hospital, Indonesia; ^2^Department of Biochemistry and Molecular Biology, Faculty of Medicine Universitas Indonesia/Cipto-Mangunkusumo Hospital, Indonesia; ^3^Division of Allergy Immunology, Department of Internal Medicine, Faculty of Medicine, Universitas Indonesia/Cipto-Mangunkusumo Hospital, Indonesia; ^4^Department of Clinical Nutrition, Universitas Indonesia/Cipto-Mangunkusumo Hospital, Indonesia; ^5^Department of Obstetrics and Gynecology, Faculty of Medicine, Universitas Padjadjaran/Dr. Hasan Sadikin General Hospital, Indonesia; ^6^Department of Obstetrics and Gynecology, Faculty of Medicine, Universitas Indonesia, Indonesia

## Abstract

**Introduction:**

Senescent cells have been demonstrated to release High Mobility Group Box 1 (HMGB1) which induces labor through an inflammatory pathway. This research is aimed at demonstrating whether telomere shortening, proinflammatory HMGB1, and oxidative damage marker 8-OHdG play a role in the placenta of preterm birth in comparison to term birth.

**Method:**

A cross-sectional study on 67 full thickness of the placenta obtained from mothers with term and preterm birth. Mothers with clinical signs of infection (fever > 38°C, leukocytosis > 18000/*μ*L, or abnormal vaginal discharge) and other pregnancy complications were excluded. Real-time polymerase chain reaction was performed to measure T/S ratio and ELISA quantification to measure the amount of HMGB1 and 8-OHdG.

**Result:**

A total of 34 placentas from preterm and 33 placentas from term birth were examined. Maternal characteristics were comparable between the two groups. There were no statistical difference of T/S ratio (*p* = 0.181), HMGB1 (*p* = 0.119), and 8-OHdG (*p* = 0.144) between the preterm and term groups. HMGB1 was moderately correlated with 8-OHdG (*r* = 0.314). Telomere T/S ratio of the placenta did not differ between preterm and term labor despite difference in gestational age, suggesting earlier shortening in the preterm group. It is possible that critical telomere length has been achieved in both term and preterm placenta that warrants labor through senescence process. The result of our study also showed that HMGB1 was not correlated to telomere length, due to the fact that HMGB1 is not upregulated until the critical length of telomere for senescence is exhibited.

**Conclusion:**

Similar telomere length might be exhibited due to early telomere shortening in preterm birth that mimics the term placenta. The relationship between placental telomere shortening and HMGB1 release remains to be uncovered. Further research is needed to discover the factors leading to early telomere shortening in the placenta of preterm birth.

## 1. Introduction

Preterm labor, defined as labor happening prior to 37 weeks of gestation, is associated with high neonatal mortality and morbidity. In 2018, globally, 15 million babies were born preterm despite advancing research and technology. Indonesia is ranked 5^th^ among 10 countries with the highest number of preterm births (675,700) and 9^th^ out of 10 countries with the highest number of preterm births for every 100 live births (15.5%) [1]. Current hypothesis suggests that inflammation plays a main role in the common pathway leading to spontaneous preterm labor [2]. Increasing evidence on sterile inflammation in the absence of infection is associated with preterm labor [3].

The role of senescence in preterm labor has been proposed [4]. The premature decidual senescence can lead to implantation failure, fetal death, and preterm birth. Recent studies have reported decidual senescence to play a role in spontaneous labor. Senescence is typically associated with shortened telomeres [5]. Fetal cellular senescence in normal pregnancy occurs physiologically, particularly at term, contributing to spontaneous parturition. Senescence may increase oxidative stress originating from the growing fetus, stretching the uterus or other unknown sources [6]. Menon compared histological evidence and biochemical markers on fetal membranes in term, preterm, and preterm pregnancy rupture of the membrane (pPROM) and found that all were positive markers of cellular senescence p53, p21, and p38MAPK. This finding suggests the role of cellular senescence in the initiation of parturition [7].

High oxidative stress leads to DNA damage shown by increased 8-hydroxy-2′-deoxyguanosine (8-OHdG) which may induce senescence [4, 8]. The higher 8-OhdG level in the placenta has previously been linked to other pregnancy complications, namely, preeclampsia, intrauterine growth retardation [9], and fetal death [10].

High Mobility Group Box 1 (HMGB1) functions as a proinflammatory mediator in response to infection and stress. Senescent amniotic membrane HMGB1 expression is possibly linked to preterm birth and pPROM [11]. Studies have shown that HMGB1 increases secretion of IL-1*β* and IL-6 and activates matrix metalloproteinase (MMP9) and collagen remodeling of chorioamnionitis membranes [12]. The evidence on HMGB1 injection into the amniotic fluid of pregnant mice has proven its role in inducing the labor process in preterm pregnant mice [13].

Research on the etiology of preterm labor had extensively focused on infection, which unfortunately does not decrease the rate of preterm birth. This study explores at other preterm pathomechanisms besides infection. Currently, no data are available on placental expression of proinflammatory HMGB1, oxidative damage marker 8-OHdG, and senescence marker telomere length in spontaneous preterm birth in the absence of infection. We aim to elucidate whether proinflammatory HMGB1 and oxidative damage marker 8-OHdG are upregulated in the placenta obtained from preterm birth and whether they are correlated with telomere shortening in inducing spontaneous preterm birth.

## 2. Methods

### 2.1. Human Ethics and Sample Collection

We conducted a cross-sectional study at Cipto Mangunkusumo Hospital and Budi Kemuliaan Hospital, Jakarta, Indonesia, from February to August 2019. This study obtained ethical approval from the ethical committee of Faculty of Medicine Universitas Indonesia ethical approval number KET-126/UN2.F1/ETIK/PPM.00.02/2019, protocol number 20-06-0624.

A total of 68 samples were recruited consecutively following informed consent. Inclusion criteria were women with singleton pregnancy presenting with labor. Term labor was defined as delivery at ≥37 weeks of gestation, otherwise preterm. Gestational age was calculated based on the last menstrual period, further confirmed by the 1st trimester ultrasound record. Preterm labor was defined as signs of labor (regular uterine contraction and cervical effacement and/or dilation) upon presentation, with <37 weeks of gestation. Women with multiple pregnancy, intrauterine growth restriction, congenital malformation, premature rupture of the membrane, maternal hypertension, or diabetes were excluded from this study. Patients with clinical signs of infection (fever > 38°C, leukocytosis > 18000/*μ*L, or abnormal vaginal discharge) were also excluded. We documented maternal characteristics and routine laboratory results to further control confounding factors.

Sample collection and handling have been described in the previous study [14]. Approximately 10 g of placental tissue was collected from 4 different sections. Placental tissue samples were taken at the full thickness of the placental parenchyma. To ensure sample stability, placenta specimen was immediately collected following delivery under sterile conditions, transported to the Prodia research laboratory for tissue sample extraction under 1 hour. Samples were stored in PBS at -70°C prior to further processing without snap freezing process.

### 2.2. Sample Processing

Placental tissue was thawed at room temperature just prior to processing. Visible villi were removed from the samples. Samples were rinsed three times with Phosphate-Buffered Saline (PBS) pH 7.0 and then dried with Whatman® filter paper. 2 mg of tissue was weighed and cut into small pieces. 1000 *μ*L of PBS was added into the samples. Samples were then placed into Precellys tissue homogenizing tubes (Bertin Technologies, France). Samples were homogenized using Precellys 24-Dual Homogenizer (Bertin Technologies, France) three times for 2 × 11 seconds at 5000 rpm speed with 10 minutes pause between the homogenization steps. Sample tubes were placed on ice during each pause to avoid overheating.

100 mg of tissue homogenates was used for DNA extraction. The rest was lysed using an ultrasonicator for 30 minutes. Samples were then centrifuged at 8500 for 15 minutes. The supernatant was collected for determination of total protein, 8-OHdG, and HMBG1 concentration. Dilution was applied prior to each analysis if required.

### 2.3. Total Protein, 8-OHdG, and HMGB1 Measurement

Total protein concentration was measured by the Bradford method using commercially available dye reagent (Bio-Rad Protein Assay, Bio-Rad, USA). 8-OHdG concentration was measured using a commercially available ELISA kit (OXIS Aoxre, Burlingame, USA), Cat No. 21026. HMGB1 concentration was measured using a commercially available ELISA kit (IBL International, Hamburg, Germany), Cat No. ST51011.

### 2.4. DNA Extraction and Telomere Length Measurement

100 mg of tissue homogenates was used for DNA extraction. DNA was extracted from placental tissues using High Pure PCR Template Preparation Kit (Cat. 11796828001, Roche Diagnostics GmbH, Germany) according to the manufacturer's protocols for isolation of nucleic acids from mammalian tissue.

### 2.5. Statistical Analysis

The minimal sample size for unpaired numerical comparative analysis was 21 for each group for two-tailed hypothesis, while the minimal sample size to analyze numerical correlation between variables was 31 for each group. Effect size for comparative analysis was set as 2, and standard error was also set as 2. Effect size for correlation analysis was set as 0.5. Significance was set at 5%, and power was set at 90%. Samples with any missing data were excluded from the analysis. Data were analyzed using the SPSS IBM version 20.0 (IBM Corp., Armonk, USA). Data were considered as homogenous if the coefficient of variation (COV) ≤ 33% and are presented as mean ± standard deviation (SD), otherwise median (interquartile range). Numerical analysis was performed using independent sample *T*-test or Mann–Whitney *U* test accordingly. Correlation was analyzed using Pearson and Spearman analysis. Preterm birth was classified further for subgroup analysis by birth before certain gestational age: early preterm group (<32 weeks) and moderate-late preterm group (32–<37 weeks) [15].

## 3. Result

A total of 67 samples were collected, 34 in the term group and 34 in the preterm group. Characteristics of the subject are presented in Table 1. The median leukocyte count was 12300 (9300–16000) *μ*L for the term group and 15100 (11355–17100) *μ*L for the preterm group (Mann–Whitney *U* test, *p* = 0.001). Other maternal characteristics were comparable between the two groups.

**Figure 1:**
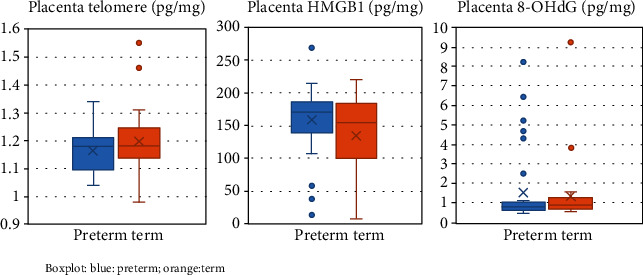
Markers of placental senescence and oxidative damage in the term and preterm groups. Blue: preterm; orange: term.

The distribution of data is shown in Figure 1. There was no difference in placental telomere ratio, 8-OhdG, and HMGB1 between the two groups (Table 2). Telomere ratios were not correlated to gestational age at labor (Pearson's correlation 0.238, *p* = 0.053) for all subjects. Further correlation analysis on telomere ratio showed no correlation with gestational age for the term and preterm groups (Pearson's correlation 0.222, *p* = 0.207 and 0.232, *p* = 0.193, respectively). HMGB1 was moderately correlated with 8-OHdG (Spearman's correlation 0.314, *p* = 0.009). Placental 8-OHdG and telomere ratio, as well as HMGB1 and telomere ratio, were not significantly correlated (*p* = 0.542 and *p* = 854, respectively).

Subgroup analysis on the preterm subject also found no statistical difference in terms of 8-OHdG, HMGB1, and telomere ratio between the early preterm and moderate-late preterm groups (Table 3). In addition, there was no correlation between gestational age with telomere length among all subjects (*n* = 68; Pearson's correlation 0.238, *p* = 0.053), the term group (*n* = 34; Pearson's correlation 0.222, *p* = 0.207), and the preterm group (*n* = 33; Pearson's correlation 0.232, *p* = 0.193).

## 4. Discussion

This study demonstrates that markers of oxidative damage (8-OHdG), proinflammatory HMGB1, and senescence (telomere) are expressed comparably between the term and preterm groups. Even so between preterm subgroups, these markers show comparable results. We also found a significant correlation between 8-OHdG and HMGB1, but neither correlates with the telomere ratio.

Shortening of telomere length (TL) has been linked to senescence; TL is influenced by DNA repair mechanisms, oxidative stress, and inflammation. TL tends to be highly correlated across tissues at birth, but this correlation diminishes as individuals age [16]. As human pregnancy progresses, telomerase activity in the placenta decreases, which in turn contributes to placental aging. Placental TL shortens during gestation, with the shortest TL exhibited at term pregnancy [17, 18]. Oxidative stress can activate the DNA damage response in the telomere and accelerate telomere shortening. In normal pregnancy, labor oxidative stress peaks at term because the increased metabolic activity of the mature fetus can produce a phenotype of the aging placenta. The risk factor for preterm delivery causes oxidative stress which triggers the breakage of single and multiple DNA strands, resulting in shortening of telomere which is not suitable for gestational age [19].

Our data reveals that earlier telomere shortening occurs in preterm labor, as shown by the equal telomere T/S ratio of the placenta from preterm and term labor. Senescence occurs when the telomere reaches its critical length that stopped the cell from further division [5, 16]. Given the similar telomere length between the preterm and term groups, it is possible that critical telomere length has been achieved in both term and preterm placenta that warrants labor. This is in conjunction with a previous study that investigates DNA damage-induced telomere attrition that releases HMGB1 in the parturition process [20]. Telomere attrition that results in shortened telomere has been implicated in other obstetrical complications, including preeclampsia and IUGR [21]. Factors leading to premature telomere shortening remain an interesting research area in the future.

HMGB1 from the placenta of both preterm and term labor has no significant difference despite the absence of intrauterine infection. Subgroup analysis of preterm birth occurring before or after 33 weeks of gestational age also reveals no significant difference between these groups. The labor process begins when there is a switch from uterine quiescent to contractile state which is marked by a proinflammatory condition, such as infection or sterile inflammation [2]. A study by Baumbusch et al. demonstrates that increased concentration of amniotic HMGB1 is parallel to increased severity of intra-amniotic inflammation and interleukin-6 [22]. Apart from infection, a previous study by Menon on placental senescence demonstrates that HMGB1 activates DAMP which upregulates expression of prolabor genes in normal parturition [7]. Thus, it can be inferred from our study that a certain level of HMGB1 that triggers labor in term pregnancy also occurs in preterm labor.

However, HMGB1 was not correlated to the telomere ratio in both groups. The causative role of HMGB1 in inducing spontaneous preterm labor and birth has been previously reported in the animal model. Our study is concurrent with this finding in terms of high HMGB1 in spontaneous preterm labor, comparable to term labor. However, we show that HMGB1 is not correlated to telomere length. We propose that this is because HMGB1 is not upregulated until the critical length of telomere for senescence is exhibited. However, unlike in term labor where the increase in HMGB1 is due to senescence-induced inflammation [7], other factors might play a role in preterm labor.

The marker of oxidative DNA damage 8-OHdG shows a similar quantity in both groups. Previous findings have shown that HMGB1 is increased in oxidative DNA stress [11], which may explain the significant correlation between HMGB1 and 8-OHdG. 8-OHdG does not show correlation with telomere length despite previous claims that DNA damage induces premature senescence [4]. Therefore, it is important to further investigate other factors, such as nutrition or environmental stress, that may affect the rate of telomere attrition and thus premature senescence in future studies.

## 5. Conclusion

Similar level of T/S ratio, HMGB1, and 8-OHdG is observed in placental sample from preterm and term birth despite difference in gestational age. Similar TL might be exhibited due to early telomere shortening in preterm birth that mimics the TL of term placenta. However, causative effect within the variables remains debatable. In addition, there was no correlation between 8-OHdG and telomere length. Further research is needed to discover the factors leading to early telomere shortening in the placenta of preterm birth.

## Figures and Tables

**Table 1:** Distribution of subject characteristics.

Characteristics	Term (*n* = 34)	Preterm (*n* = 33)	*p* value
Age (years)	29.59 ± 5.79	27.79 ± 7.36	0.288
Nullipara/multipara (%)^#^	12 (17.91%)/22 (32.84%)	20 (29.85%)/14 (20.90%)	0.088
Preterm history (%)^#^	2 (2.99%)	2 (2.99%)	1.000
Maternal BMI (kg/m^2^)	22.39 ± 2.51	21.28 ± 2.98	0.103
Maternal UAC (cm)	26.80 ± 3.62	25.59 ± 3.43	0.160
Hemoglobin (g/dL)	10.92 ± 1.50	11.47 ± 1.35	0.121
Leukocyte (/*μ*L)^	10200 (8975–13125)	15100 (11355–17100)	0.001^∗^
Thrombocyte (10^3^/*μ*L)	259.18 ± 70.98	271.00 ± 64.90	0.476
Gestational age at labor (week)	39.44 ± 1.13	32.53 ± 2.57	<0.001^∗^
Birth weight (gram)	3176.71 ± 400.07	1884.56 ± 542.60	<0.001^∗^
Birth length (cm)	48.94 ± 1.98	42.31 ± 4.55	<0.001^∗^
Placental weight (gram)	511.50 ± 78.36	407.24 ± 99.18	<0.001^∗^
Spontaneous labor/vacuum assisted/caesarean section (%)^#^	13 (19.40%)/3 (4.48%)/18 (26.87%)	25 (37.31%)/0 (0%)/8 (11.94%)	0.002^∗^

Homogenous data presented as mean ± SD, the *p* value for independent sample *T*-test; ^#^frequency (percentage from total), chi-square or Fisher's exact; ^median (interquartile range), Mann–Whitney *U* test; ^∗^*p* value < 0.05. SDB: systolic blood pressure; DBP: diastolic blood pressure; BMI: body mass index; UAC: upper arm circumference; OGTT: oral glucose tolerance test.

**Table 2:** Markers of placental senescence and oxidative damage in the term and preterm groups.

Markers	Preterm (*n* = 33)	95% CI of mean	Term (*n* = 34)	95% CI of mean	*p* value
Telomere ratio T/S	1.164 ± 0.078	1.137–1.192	1.196 ± 0.112	1.157–1.235	0.181
HMGB1 (pg/mg)^	166.12 (137.88–186.44)	140.41–176.58	154.39 (100.26–183.69)	113.19–156.93	0.119
8-OHdG (pg/mg)^	0.771 (0.631–1.048)	0.874–2.241	0.883 (0.703–1.2560)	0.794–1.905	0.144

mean ± SD, independent sample *T*-test; ^median (interquartile range), Mann–Whitney *U* test; ^∗^*p* value < 0.05.

**Table 3:** Markers of placental senescence and oxidative damage within preterm subgroups.

Markers	Very preterm (*n* = 11) (<32 weeks)	95% CI of mean	Late preterm (*n* = 22) (32–<37 weeks)	95% CI of mean
Telomere ratio T/S	1.145 ± 0.049	1.113–1.178	1.174 ± 0.089	1.134–1.213
HMGB1 (pg/mg)^	165.57 (132.20–192.66)	137.73–199.14	170.75 (140.03–185.34)	129.69–177.36
8-OHdG (pg/mg)^	0.657 (0.627–0.935)	0.307–2.228	0.808 (0.635–1.089)	0.748–2.656

mean ± SD, independent sample *T*-test; ^median (interquartile range), Mann–Whitney *U* test; ^a^*p* value of very preterm vs. term < 0.05; ^b^*p* value of late preterm vs. term < 0.05; ^c^*p* value of very vs. late preterm <0.05.

## Data Availability

The datasets analyzed in the study are available from the corresponding author upon request.

## References

[1] World_Health_Organization (2018). Preterm birth. https://www.who.int/news-room/fact-sheets/detail/preterm-birth.

[2] Romero R., Dey S. K., Fisher S. J. (2014). Preterm labor: one syndrome, many causes. *Science*.

[3] Romero R., Miranda J., Chaiworapongsa T. (2014). Prevalence and clinical significance of sterile intra-amniotic inflammation in patients with preterm labor and intact membranes. *American Journal of Reproductive Immunology*.

[4] Sultana Z., Maiti K., Dedman L., Smith R. (2018). Is there a role for placental senescence in the genesis of obstetric complications and fetal growth restriction?. *American journal of obstetrics and gynecology*.

[5] Bernadotte A., Mikhelson V. M., Spivak I. M. (2016). Markers of cellular senescence. Telomere shortening as a marker of cellular senescence. *Aging*.

[6] Menon R. (2016). Human fetal membranes at term: dead tissue or signalers of parturition?. *Placenta*.

[7] Menon R. (2019). Initiation of human parturition: signaling from senescent fetal tissues via extracellular vesicle mediated paracrine mechanism. *Obstet Gynecol Sci.*.

[8] Dabrowska N., Wiczkowski A. (2017). Analytics of oxidative stress markers in the early diagnosis of oxygen DNA damage. *Advances in Clinical and Experimental Medicine*.

[9] Londero A. P., Orsaria M., Marzinotto S. (2016). Placental aging and oxidation damage in a tissue micro-array model: an immunohistochemistry study. *Histochemistry and Cell Biology*.

[10] Maiti K. S. Z., Aitken R. J., Morris J. (2017). Evidence that fetal death is associated with placental aging. *American Journal of Obstetrics and Gynecology*.

[11] Bredeson S., Papaconstantinou J., Deford J. H. (2014). HMGB1 promotes a p38MAPK associated non-infectious inflammatory response pathway in human fetal membranes. *PLoS One*.

[12] Plazyo O. (2016). The role Of alarmins, invariant Nkt cells and senescence in the pathophysiology of sterile intra-amniotic inflammation. *Wayne State University*.

[13] Gomez-Lopez N., Romero R., Plazyo O. (2016). Intra-amniotic administration of HMGB1 induces spontaneous preterm labor and birth. *American Journal of Reproductive Immunology*.

[14] Irwinda R., Wibowo N., Putri A. S. (2019). The concentration of micronutrients and heavy metals in maternal serum, placenta, and cord blood: a cross-sectional study in preterm birth. *Journal of Pregnancy*.

[15] Quinn J. A., Munoz F. M., Gonik B. (2016). Preterm birth: case definition & guidelines for data collection, analysis, and presentation of immunisation safety data. *Vaccine*.

[16] Jones C. W., Gambala C., Esteves K. C. (2017). Differences in placental telomere length suggest a link between racial disparities in birth outcomes and cellular aging. *American Journal of Obstetrics and Gynecology*.

[17] Kohlrausch F. B., Keefe D. L. (2020). Telomere erosion as a placental clock: from placental pathologies to adverse pregnancy outcomes. *Placenta*.

[18] Gielen M., Hageman G., Pachen D., Derom C., Vlietinck R., Zeegers M. P. (2014). Placental telomere length decreases with gestational age and is influenced by parity: a study of third trimester live-born twins. *Placenta*.

[19] Ferrari F., Facchinetti F., Saade G., Menon R. (2016). Placental telomere shortening in stillbirth: a sign of premature senescence?. *The Journal of Maternal-Fetal & Neonatal Medicine*.

[20] Menon R., Behnia F., Polettini J., Saade G. R., Campisi J., Velarde M. (2016). Placental membrane aging and HMGB1 signaling associated with human parturition. *Aging (Albany NY)*.

[21] Niu Z., Li K., Xie C., Wen X. (2019). Adverse birth outcomes and birth telomere length: a systematic review and meta-analysis. *The Journal of Pediatrics*.

[22] Baumbusch M. A., Buhimschi C. S., Oliver E. A. (2016). High mobility group-box 1 (HMGB1) levels are increased in amniotic fluid of women with intra-amniotic inflammation-determined preterm birth, and the source may be the damaged fetal membranes. *Cytokine*.

